# Supplementary material for: Mortality in ICU Patients with COVID-19-Associated Pulmonary Aspergillosis
Source: J Fungi (Basel). 2023 Jun 20;9(6):689. doi: 10.3390/jof9060689 (PMC10305703; doi:10.3390/jof9060689)
Supplement: Supplementary file 1 [file jof-09-00689-s001.zip › jof-2392946-supplementary.pdf]

Journal of *Fungi*

MDPI

Supplementary material

Mortality in ICU Patients with COVID-19-Associated Pulmonary Aspergillosis

Table S1. Comorbidities of 1,437 CAPA patients included in the review.

Author, [reference number] publication's year	Comorbidities
Araya-Rojas[1], 2021	Chronic kidney disease 9.1%<br>Chronic obstructive pulmonary disease 9.1%<br>Diabetes mellitus 63.6%<br>Hypertension 45.4%<br>Neutropenia 9.1%<br>Obesity 63.6%
Bartoletti[2], 2021	Cerebrovascular disease 10%<br>Chronic kidney disease 20%<br>Chronic obstructive pulmonary disease 13%<br>Coronary disease 10%<br>Diabetes mellitus 17%<br>Hypertension 59%<br>Malignancies 7%<br>Obesity 37%<br>Solid organ transplant 3%
Bentvelsen[3], 2022	Chronic obstructive pulmonary disease 25%<br>Immunocompromised 4.4%<br>Obesity 28.1%<br>Pulmonary disease 59.1%
Bretagne[4], 2021	NA
Calderon-Parra[5], 2022	Chronic cardiac failure 21.4%<br>Chronic renal failure 25%<br>Chronic respiratory disease 48.1%<br>Diabetes mellitus 39.3%<br>Hematologic cancer 14.3%<br>Hypertension 64.3%<br>Immunocompromised 42.9%<br>Ischemic heart disease 21.4%<br>Liver cirrhosis 7.1%<br>Solid malignancy 7.1%<br>Solid organ transplant 17.9%
Casalini[6], 2022	Cardiovascular disorders 57.1%<br>Chronic kidney disease 4.2%<br>Diabetes 17.3%<br>Hepatic disorders 2.4%<br>Immune system disorders 4.2%<br>Obesity 35.7%

	Oncological disorders 8.3%<br>Respiratory disorders 10.1%
De Almeida[7],<br>2022	Diabetes 57%<br>Heart disease 36%<br>Hypertension 50%<br>Cancer 21%<br>Chronic renal failure 7%<br>Chronic obstructive pulmonary disease 36%
Delliere[8],<br>2020	Asthma 9.5%<br>Chronic obstructive pulmonary disease 0<br>Coronary disease 9.5%<br>Diabetes 42.9%<br>Hypertension 66.7%<br>Immunocompromised 9.5%<br>Obesity 19%
Dupon[9],<br>2021	Asthma 21.1%<br>Chronic obstructive pulmonary disease 21.1%<br>Chronic renal failure 10.5%<br>Diabetes 36.8%<br>Hypertension 36.8%<br>Obesity 5.3%<br>Solid cancer 15.8%
Er[10],<br>2022	Asthma 4.7%<br>Chronic obstructive pulmonary disease 9.3%<br>Chronic renal failure 18.6%<br>Collagen tissue disease 2.3%<br>Congestive heart failure 23.3%<br>Coronary artery disease 30.2%<br>Diabetes mellitus 37.2%<br>Hypertension 46.5%<br>Solid organ transplantation 2.3%<br>Solid organ tumor 23.3%
Erami[11],<br>2022	Acute lymphoblastic leukemia 5.9%<br>Acute myeloblastic leukemia 11.7%<br>Chronic myeloblastic leukemia 5.9%<br>Diabetes mellitus 70.6%<br>Heart failure 29.4%<br>Kidney disorder 35.3%<br>Liver transplantation 17.6%
Ergun[12],<br>2021	Autoimmune disease 5.1%<br>Cardiovascular disease 41%<br>Chronic kidney disease 2.6%<br>Diabetes mellitus 25.6%<br>Hematological malignancy 2.6%<br>Pulmonary disease 23.1%<br>Solid organ malignancy 10.3%<br>Solid organ transplant 2.6%<br>Stem cell transplant 2.6%
Fischer[13],	Asthma 0

2022	Autoimmune disease 0<br>Cardiovascular disease 46.2%<br>Cerebrovascular disease 30.8%<br>Chronic obstructive pulmonary disease 15.4%<br>Diabetes 30.8%<br>Hematologic disease 7.7%<br>Hypertension 61.5%<br>Neoplasm 7.7%<br>Other pulmonary disease 0
Fortun[14],<br>2023	Active hematologic disorder 8%<br>Active solid tumor 5.8%<br>Chronic renal failure 10.9%<br>Diabetes mellitus 26.8%<br>Hepatic insufficiency (acute or chronic) 10.9%<br>Progenitor hematologic transplant 2.9%<br>Renal insufficiency (acute or chronic) 35%<br>Solid organ transplant 1.4%
Gangneux[15],<br>2022	Asthma 7%<br>Chronic obstructive pulmonary disease 11%<br>Diabetes 34%<br>Dyslipidemia 3%<br>Hypertension 47%<br>Immunodepression 14%<br>Solid organ transplantation 11%
Giacobbe[16],<br>2022	NA
Giusiano[17],<br>2022	Chronic obstructive pulmonary disease 10.5%<br>Diabetes 31.6%<br>Emphysema 5.3%<br>HIV infection 5.3%<br>Hypertension 47.4%<br>Lymphoma 5.3%<br>Obesity 26.3%<br>Renal insufficiency 5.3%<br>Ulcerative colitis 5.3%<br>Vasculitis 5.3%
Hashim[18],<br>2022	Chronic kidney disease 7.7%<br>Chronic liver disease 5.4%<br>Chronic pulmonary disease 23%<br>Coronary artery disease 7.7%<br>Diabetes mellitus 60.3%<br>HIV infection 1.4%<br>Hypertension 45.9%<br>Malignancy 2.7%<br>Renal transplantation 2.7%
Hatzl[19],<br>2021	NA
Huang[20],<br>2022	Chronic airway diseases 0<br>Chronic kidney diseases 27%<br>Cirrhosis 9%<br>Diabetes mellitus 45%

	Malignancies 9%
Iqbal[21], 2021	Diabetes mellitus 73.8%<br>Cirrhosis 8.2%<br>Hypertension 49.2%<br>Malignancy 21.3%
Janssen[22], 2021	Asthma 2%<br>Cardiovascular disease 60%<br>Chronic obstructive pulmonary disease 19%<br>Diabetes mellitus 21%<br>HIV/AIDS 7%<br>Liver cirrhosis 0<br>Rheumatological disease 12%<br>Solid organ malignancy 7%
Kim[23], 2022	Diabetes mellitus 23.5%<br>Hypertension 64.7%<br>Chronic heart disease 11.8%<br>Chronic lung disease 29.4%<br>Chronic kidney disease 0<br>Malignancy 5.9%<br>Chronic hepatitis 0<br>Neurologic disease 5.9%
Koukaki[24], 2022	Chronic respiratory diseases 14.3%<br>Diabetes 14.3%<br>Hypertension 35.7%<br>Hypothyroidism 7.1%<br>Immunosuppression 21.4%
Lahmer[25], 2021	Asthma 0<br>Atrial fibrillation 9%<br>Chronic obstructive pulmonary disease 18%<br>Coronary heart disease 9%<br>Chronic kidney disease 9%<br>Diabetes mellitus 27%<br>Hypertension 63%
Lee[26], 2022	Chronic kidney disease 20%<br>Chronic liver disease 20%<br>Chronic obstructive pulmonary disease 20%<br>Diabetes mellitus 50%<br>Heart failure 10%<br>Hematologic malignancies 20%<br>History of hematopoietic stem cell transplantation 10%<br>History of solid organ transplantation 0<br>Solid cancer 10%
Leistner[27], 2022	AIDS/HIV 0<br>Cancer 11%<br>Diabetes mellitus 32%<br>Heart disease 15%<br>Liver disease 23%<br>Lung disease 40%<br>Peptic ulcer 4%<br>Renal disease 87%<br>Rheumatic disease 4%

	Neurologic disease 6%<br>Vascular disease 17%
Marta[28],<br>2022	Cardiovascular disease 31.4%<br>Chronic pulmonary disease 22.8%<br>Diabetes mellitus 25.7%<br>Dyslipidemia 51.4%<br>Hypertension 65.7%<br>Obesity 34.3%
Melchers[29],<br>2022	Active oncologic disease 15%<br>Cardiac disease 62%<br>Chronic respiratory disease 31%<br>Diabetes mellitus 62%<br>Renal insufficiency 15%
Permpalung[30],<br>2022	Alcohol abuse 2.6%<br>Anemia, blood loss 10.3%<br>Anemia, nutritional deficiency 71.8%<br>Chronic pulmonary disease 41%<br>Coagulopathy 51.3%<br>Congestive heart failure 46.2%<br>Depression 30.8%<br>Diabetes mellitus complicated 38.5%<br>Diabetes mellitus uncomplicated 38.5%<br>Drug abuse 7.7%<br>Fluid and electrolytic disorders 97.4%<br>HIV/AIDS 2.6%<br>Hypertension 74.4%<br>Hypertension complicated 56.4%<br>Hypothyroidism 10.3%<br>Liver disease 35.9%<br>Lymphoma 2.6%<br>Monoclonal gammopathy 0<br>Multiple myeloma 5.1%<br>Obesity 41%<br>Other neurologic disorders 53.8%<br>Paralysis 17.9%<br>Peptic ulcer disease excluding bleeding 7.7%<br>Peripheral vascular disorders 15.4%<br>Pulmonary vascular diseases 41%<br>Psychosis 12.8%<br>Renal failure 43.6%<br>Rheumatoid arthritis/collagen vascular diseases 2.6%<br>Solid organ transplant 0<br>Solid tumor 25.6%<br>Weight loss 35.9%
Prattes[31],<br>2022	Active malignant disease 10.3%<br>Cardiovascular disease 57.8%<br>Diabetes mellitus 29.4%<br>History of smoking 13.3%<br>Obesity 28.2%<br>Pulmonary disease 23.9%

	Solid organ transplantation 4.9%
Ranhel[32],<br>2021	Alzheimer's disease 10%<br>Atrial fibrillation 10%<br>Chronic obstructive pulmonary disease 30%<br>Diabetes mellitus 20%<br>Dyslipidemia 30%<br>Hypertension 50%<br>Hypothyroidism 30%<br>Mono nephrectomy 10%<br>Nephropathy 10%<br>Obesity 20%<br>Osteoporosis 20%<br>Psoriasis 10%<br>Solid organ transplantation 30%<br>Sjogren syndrome 10%
Rouze[33],<br>2022	Diabetes mellitus 28.6%<br>Chronic heart failure 0<br>Chronic kidney disease 7.1%<br>Chronic respiratory failure 7.1%<br>Cirrhosis 0<br>Chronic obstructive pulmonary disease 14.3%<br>Heart disease 14.3%<br>Hematologic malignancy 7.1%<br>HIV 0<br>Immunosuppression 14.3%<br>Immunosuppressive drugs 14.3%<br>Organ transplant 0<br>Solid cancer 0
Sivasubramanian[34],<br>2021	Coronary artery disease 35%<br>Diabetes mellitus 64%<br>Hypertension 79%<br>Liver disease 12%<br>Lung disease 25%<br>Obesity 45%<br>Renal disease 25%<br>Stroke/dementia 22%
Velez Pintado[35],<br>2021	Cancer 19%<br>Chronic obstructive pulmonary disease 6%<br>Diabetes mellitus 31%<br>Hypertension 25%<br>Obesity 38%<br>Overweight 50%
White[36],<br>2020	Alzheimer's disease 4%<br>Autoimmune disease 8%<br>Cardiac disease 4%<br>Chronic kidney disease 8%<br>Chronic respiratory illness 48%<br>Diabetes mellitus 24%<br>Essential thrombocythemia 4%<br>Hematologic malignancy 4%<br>Hypertension 32%

	Liver disfunction 4%<br>Obesity 24%<br>Solid cancer 20%<br>Vasculitis 4%
Xu[37],<br>2021	Autoimmune disease 3.3%<br>Cerebrovascular disease 12.8%<br>Chronic cardiac disease 16.7%<br>Chronic kidney disease 3.9%<br>Chronic liver disease 6.4%<br>Chronic pulmonary disease 6.4%<br>Coronary disease 10.3%<br>Diabetes mellitus 48.7%<br>Hypertension 48.7%<br>Liver cirrhosis 1.3%
Zhang[38],<br>2021	Asthma 18%<br>Chronic obstructive pulmonary disease 24%<br>Diabetes mellitus 36%<br>End stage renal disease 12%<br>Hyperlipidemia 21%<br>Hypertension 58%<br>Obesity 21%
